# High-intensity Fitness Training Among a National Sample of Male Career Firefighters

**DOI:** 10.1016/j.shaw.2014.12.005

**Published:** 2015-01-12

**Authors:** Sara A. Jahnke, Melissa L. Hyder, Christopher K. Haddock, Nattinee Jitnarin, R. Sue Day, Walker S. Carlos Poston

**Affiliations:** 1 Center for Fire, Rescue, and EMS Health Research, Institute for Biobehavioral Health Research, National Development and Research Institutes, Leawood, KS, USA; 2 University of Texas Health Science Center at Houston, Houston, TX, USA

**Keywords:** firefighter, fitness, high intensity training

## Abstract

Obesity and fitness have been identified as key health concerns among USA firefighters yet little is known about the current habits related to exercise and diet. In particular, high-intensity training (HIT) has gained increasing popularity among this population but limited quantitative data are available about how often it is used and the relationship between HIT and other outcomes. Using survey methodology, the current study evaluated self-reported HIT and diet practice among 625 male firefighters. Almost one-third (32.3%) of participants reported engaging in HIT. Body composition, as measured by waist circumference and percentage body fat, was significantly related to HIT training, with HIT participants being approximately half as likely to be classified as obese using body fat [odds ratio (OR) = 0.52, 95% confidence interval (CI) = 0.34–0.78] or waist circumference (OR = 0.61, 95% CI = 0.37–0.98). Those who engaged in HIT were more than twice as likely as those who did not (OR = 2.24, 95% CI = 1.42–3.55) to meet fitness recommendations. Findings highlight directions for future prevention and intervention efforts.

## 1 Introduction

Firefighting is an inherently dangerous, physically demanding occupation requiring adequate levels of fitness to fulfill mandatory job duties. Physically taxing activities performed by firefighters may include hauling hose, using heavy extrication equipment, or lifting and transporting patients [1–3]. Despite the need for high levels of readiness and fitness, recent research suggests high rates of obesity among firefighters. Rates of overweight and obesity in the fire service (estimated at about 80% combined) [4,5] are higher than those found among the general United States (US) public (68% are overweight or obese) [6]. Obesity is an even greater risk for firefighters given the duties they are required to perform at a moment's notice, often in suboptimal conditions such as heat, smoke, and chemical exposure [7].

Previous studies documented that overweight and obese firefighters have elevated rates of hypertension, low High-density lipoprotein (HDL) cholesterol, high Low-density lipoprotein (LDL) cholesterol, high triglycerides, significant yearly weight gain, lower cardiorespiratory fitness, reduced muscle strength, and more frequent fatal cardiac events [7,8]. In turn, cardiovascular disease (CVD) is the leading cause of line of duty deaths (LODDs) among firefighters, implicated in nearly half of LODDs, with the majority occurring during fire suppression activities [9]. In addition to serving as a risk factor for CVD, a study by Jahnke et al [10] found that obese firefighters were 5.2 times more likely to incur a musculoskeletal injury than their healthy weight peers. In addition, obesity has been found to be related to low rates of fitness in firefighters [4]. Evidence suggests that firefighting duties require approximately 12 metabolic equivalent units of effort (METs) [11], which is recommended by national standards as a return to work standard for firefighters following a cardiac event [12]. In a population-based sample of firefighters, nearly all of whom were obese (80% career, 95% volunteer) by body mass index (BMI) standards would not be able to meet this fitness recommendation [4]. Given the risks firefighters face and the need for high levels of fitness, high rates of overweight and obesity are of particular concern.

Given the importance of fitness for firefighters, innovative techniques for improving their overall fitness responsive to the unique training needs, culture, and environment of the fire service [1] are needed. One promising approach to promoting fitness among firefighters is high-intensity training (HIT), which is an approach to exercise including functional movements at a high level of effort, but short in duration [13]. These exercise programs may be a better way for firefighters to prepare for what their job requires because they focus on full body movements, closely mimicking actual fire ground activities [1,13]. Roberts et al [14] evaluated a 16-week HIT program for new firefighter recruits and found 3 days per week of training for 1 hour per session resulted in significant improvements in maximal oxygen consumption (VO_2max_). Training with HIT not only provides improvement in cardiorespiratory fitness but does so in less time than traditional training, and will be likely to prove beneficial for firefighters' constrained and unpredictable schedules [14].

While the fire service is working to improve health and wellness among its members, limited data are available about current fitness in general and HIT in particular that firefighters engage in. Thus, the purpose of the current study is to assess the self-reported health practices of male firefighters, specifically related to fitness and HIT. In addition, we will explore the relationship between self-reported engagement in HIT, obesity, and fitness among this important population. Findings will provide a foundation of knowledge from which to build future prevention and intervention efforts.

## 2 Materials and methods

### 2.1 Procedures

The data reported are from the follow-up evaluation of a longitudinal cohort study of firefighters examining general health and quality of life including measured height and weight, estimated percentage body fat (BF%), blood pressure, and self-reported measures of diet, physical activity, substance use, mood, sleep, injury, and job satisfaction. All data were collected between 2011 and 2013. Departments (*n* = 20) ranged in size from small (≤3 stations; *n* = 7), medium (>3 stations, but <350 personnel; *n* = 8), and large (≥350 personnel; *n* = 5) based on the fire services' classification [15]. They also encompassed different settings (4 rural, 10 suburban, 2 urban, and 4 mixed urban) and regions based on US Census regions (8 Pacific/West, 8 Mountain/Central, and 4 East/Southeast). All departments were recruited by a request for participation on The Secret List (www.firefighterclosecalls.com), which is a widely distributed listserv within the US Fire Service. Data were collected for all participants at baseline and 6-month follow-up. More detailed recruitment procedures can be found in Poston et al [16]. The protocol for the protection of participants for this study was approved by the Institutional Review Board of the National Development and Research Institutes, Inc., Leawood, KS, USA and the University of Texas Houston Health Sciences Center, Houston, TX, USA.

### 2.2 Measures

#### 2.2.1 Demographics

Standard individual (e.g., age, marital status, educational level, and race) and occupational (rank and years in the fire service) demographic data were collected using previously published questions [4].

#### 2.2.2 Body composition: weight, BMI, BF%, and waist circumference

Height was assessed by using a portable stadiometer. Body weight and BF% were assessed using foot-to-foot bioelectrical impedance (Tanita 300; Tanita Corporation of America, Inc., Arlington Heights, IL, USA). The Tanita 300 has demonstrated strong evidence of concurrent validity (r = 0.94; *p* < 0.001) [17] when compared with the “criterion standard” of dual energy X-ray absorptiometry for BF assessment. It is a commonly used bioelectrical impedance field measure because of its portability and accuracy in determining BF% [17]. Waist circumference (WC) was assessed using a spring-loaded nonstretchable tape measure as recommended by the US Government obesity guidelines [18]. Standard cut-points were used for obesity (BMI ≥ 30, BF% ≥ 25, WC ≥ 40) [18].

#### 2.2.3 HIT

Participation in HIT was assessed by asking the following question: “Do you currently base your workouts on high-intensity fitness training programs such as CrossFit, P90X, or Insanity?” Response options were: If the participant answered yes, they were asked to indicate “How long (in months) have you participated in a high-intensity training program?” and “On average, how many days a week do you do high-intensity workouts?” For analyses of HIT, participants were divided into two categories (HIT and No HIT).

#### 2.2.4 Physical activity

The self-report of physical activity (SRPA) was used to assess a global self-rating of physical activity patterns [19]. Participants selected a value (0–7) on the SRPA most closely indicating their physical activity over the past 30 days. Indicators of the questionnaire's validity in adult populations have been established (i.e., significant correlations between SRPA ratings and measured VO_2max_) [20]. Scores on the SRPA, BMI, and age were used to estimate VO_2max_ with a method developed and tested in comparison with measured VO_2max._ The method has been found to be equal or better in accuracy compared to submaximal exercise testing [20]. METs were calculated based on VO_2max_ and used to classify firefighters into those meeting the suggested minimal fitness standard of 12 METs and those who did not. The cut off is based on findings of Donovan et al [2] indicating firefighting activities require 12 METs of energy as well as National Fire Protection Association standards showing a firefighter must be able to achieve 12 METs to return to work postcardiac event [12].

### 2.3 Statistical analysis

Data are presented as mean (M) and standard deviation (SD) for continuous variables and percentages for categorical variables. Logistic models were used to determine the relationships between engaging in HIT and body composition (BMI, WC, BF%) as well as the relationship between HIT and meeting minimum fitness recommendations. Preliminary analyses revealed that those who used HIT were significantly younger than those who did not (M = 37.5 years, SD = 8.3 years and M = 40.4 years, SD = 8.9 years respectively; *t* = 3.8, *p* < 0.001). In addition, departments engaging in wellness or fitness programs have previously been found to be significantly related to body composition [16]; therefore, all HIT logistic models were controlled for department type and age. We also examined the dose-response relationship between HIT frequency and body composition among HIT users with a logistic model. Finally, to determine whether there was a difference between firefighters who practiced HIT and those who did not, we selected for those who indicated they regularly participate in heavy exercise (SRPA score of 4–7) and conducted logistic regressions examining the relationship between HIT status and body composition/fitness outcomes. Statistical analyses were conducted using IBM SPSS Statistics version 21 (SPSS Inc., Chicago, IL, USA).

## 3 Results

### 3.1 Participants

The initial sample at baseline was 1,035 firefighters. Due to the low number of women (3.2%) and the inability to make statistical inferences based on gender, they were excluded from this analysis. Of the remaining 1,002 male firefighters, 64.9% (*n* = 650) had data available at follow-up and 625 had responses to the relevant questions. Those responding to the questions did not differ significantly from those who did not with regard to baseline demographics or health parameters. The majority of participants (80.7%) were Caucasian with an average age of 39.4 (SD = 8.8) years and 14.6 (SD = 8.6) years of fire service experience (Table 1). Most firefighters (88.0%) had some college education or a college degree.

### 3.2 HIT

Nearly one-third (32.3%) of participants reported engaging in HIT. Body composition, as measured by WC and BF%, was significantly related to HIT training, with HIT participants being approximately half as likely to be classified as obese using body fat (OR = 0.52, 95%CI = 0.34–0.78) or WC (OR = 0.61, 95%CI = 0.37–0.98). The relationship was not significant when using BMI-defined obesity (OR = 0.68, 95%CI = 0.44–1.04). Those who engaged in HIT were more than twice as likely as those who did not (OR = 2.24, 95%CI = 1.42–3.55) to meet the 12 MET recommendations.

**Fig. 1:**
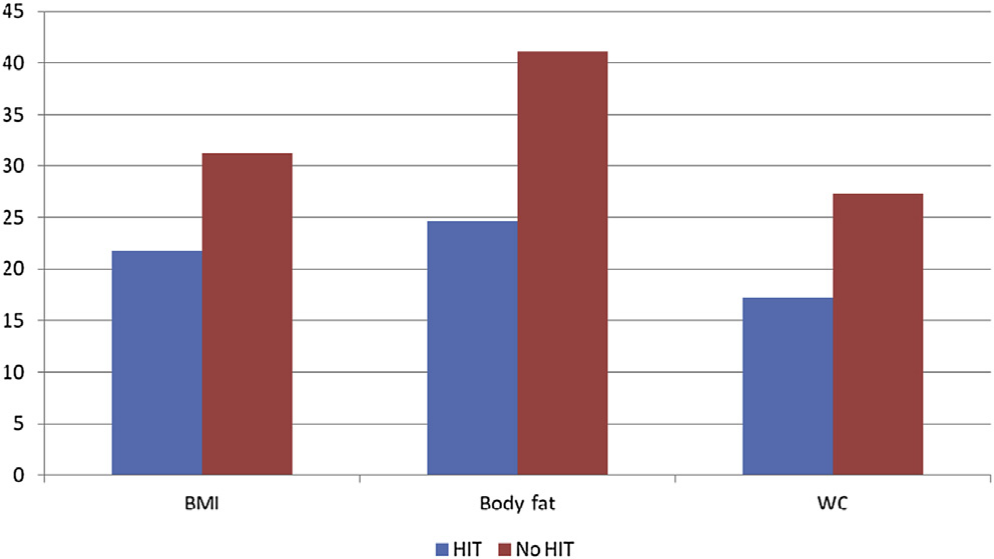
Unadjusted obesity status rate by high-intensity training (HIT).

After controlling for department type and age, both the length of time and the amount of training per week were significant predictors of body composition and fitness among those who engaged in HIT. The length of time (in months) a participant had engaged in HIT was related to improved body composition and fitness. For example, each month of reported HIT resulted in a 5–6% reduction in BMI-derived (OR = 0.95, 95%CI = 0.91–0.99) and WC-derived obesity status (OR = 0.94, 95%CI = 0.89–0.99; Fig. 1). By contrast, for every month of reported HIT training, participants were 3% more likely to meet the 12 MET requirement (OR = 1.03, 95%CI = 1.01–1.05). Days per week of engaging in HIT also was significantly related to reductions in the risk of being classified as obese based on body fat (OR = 0.74, 95%CI = 0.56–0.99) and BMI (OR = 0.71, 95% CI = 0.53–0.96). Days per week of HIT training also was significantly related to meeting the 12 MET recommendation, with each day of training being related to a doubling of chances of meeting the standard (OR = 2.00, 95%CI = 1.37–2.91).

### 3.3 SRPA and HIT

Firefighters who engaged in HIT had significantly higher scores on the SRPA than those who did not (M = 5.57, SD = 1.83 vs. M = 4.66, SD = 1.83; *t* = –5.92, *p* < 0.001). Among those who reported engaging regularly in heavy physical fitness on the SRPA, those who participated in HIT were less likely to be obese by body fat standards (OR = 0.56, 95%CI = 0.34–0.90). However, findings were not statistically significant for BMI-defined obesity (OR = 0 .92, 95%CI = 0.56–1.53) or WC-defined obesity (OR = 0.82, 95%CI = 0.47–1.43). Those who used HIT evidenced an increase in the likelihood of meeting minimal fitness standards (OR = 1.56, 95% CI = 0.92–2.63), although the finding did not reach statistical significance (*p* = 0.10).

## 4 Discussion

Approximately one-third of firefighters reported that they participated in some sort of HIT and those who did were approximately half as likely to be in the obese range by WC and BF% measures when compared to those who did not. The lack of a relationship between HIT and BMI may be due to misclassification of those with high BMI but low body fat among those whose are active. Firefighters engaging in HIT also were more likely to meet the 12 MET standard for exertion when compared to those who did not. In addition, a dose response relationship existed both between number of months and the number of days per week engaging in HIT. For days per week, each additional day engaging in HIT doubled the chances of achieving the 12 MET recommendation. Benefits of HIT in improving body composition are consistent with previous research indicating a significant relationship between HIT, fitness, and body composition [13]. Even among those who reported engaging in regular heavy exercise, those who practiced HIT were significantly less likely to be obese by body fat measures. Findings were not consistent for this group across body composition measures, probably because the study was not powered to answer this question specifically. In addition, while the magnitude of the relationship between meeting the fitness standard and HIT among this group was considerably large (OR = 1.56), it was not statistically significant, probably for the same power reasons. Overall, findings highlight the potential benefit of HIT as a training modality for firefighters.

As with any research, there are limitations to the current study. First, this was not a randomly selected sample of firefighters. Departments volunteered for the project in response to an advertisement on www.firefighterclosecalls.com both for those with and without a wellness approach. This may be a limiting factor as departments volunteering may focus more on health and wellness (or at least the desire to focus more on health and wellness). Second, due to their small numbers and the resulting lack of statistical power, women were removed from all analyses, which limits the generalizability to male firefighters. Third, because the interest was more about what choices were made while on duty and at the station, only career firefighters were included in this study. Despite limitations, there are numerous strengths of the current study. First, this was a large, national cohort of firefighters. Second, there were small, medium, and large departments in rural, suburban, and urban settings, which makes this one of the largest and most geographically diverse studies of the fire service's self-reported dietary and physical activity habits. Another strength is the objective measurement of body composition and obesity status using three methods (BMI, WC, and BF%). The current findings provide a strong basis for future research. Given the current rate of LODDs due to CVD, physical activity should continue to be areas of focus. The fire service has taken significant steps toward increasing physical fitness among firefighters with the Wellness Fitness Initiative although most departments have not adopted wellness programs. Policy change related to physical fitness is needed to keep firefighters safe and prepared for the physical demands of the job. Future research should attempt to find novel ways to decrease CHD risk factors such as obesity, while maintaining or increasing fitness, so firefighters remain prepared for duty.

## Conflicts of interest

All contributing authors declare no conflicts of interest.

## Figures and Tables

**Table 1:** Demographics of participants

Characteristic	%/mean (standard deviation)
Age (y)	39.4 (8.8)
% married	73.2
% Caucasian	80.7
Time in fire service (y)	14.6 (8.6)
Rank	
Firefighter	34.0
Firefighter/paramedic	13.6
Driver operator	19.7
Company officer (Lt., Capt.)	22.5
Chief (including Battalion, Deputy)	4.6
Other	5.6
Education	
High School	9.7
Some college or tech school	67.2
College graduate	20.8
Advanced degree	2.3
